# Prevalence of Depression among Stroke Survivors in India: A Systematic Review and Meta-Analysis

**DOI:** 10.51956/FNNR.100008

**Published:** 2021-04-14

**Authors:** Abhilash Patra, Karakapatla Nitin, Ng Marina Devi, Srinivasan Surya, Melissa G. Lewis, Sureshkumar Kamalakannan

**Affiliations:** 1 Indian Institute of Public Health, Kaloji Narayana Rao University of Health Sciences, India

**Keywords:** stroke, depression, systematic review, rehabilitation, prevalence, India

## Abstract

**Objective:**

To estimate the prevalence of depression among stroke survivors in India.

**Participants:**

Stroke survivors diagnosed with depression.

**Outcomes:**

Prevalence of Depression.

**Methods:**

Cochrane systematic review methods were followed. The literature search was from 1960-2019. We searched the following electronic databases Medline, ERIC, Embase, IndMED, PsycEXTRA, Global Health, Cochrane, CENTRAL Register, Econ Lit, and conference abstracts to identify studies for inclusion. A search strategy was appropriately developed and performed from May 2019 to December 2019. All included studies were assessed for their content and methodological quality using JBI Critical Appraisal Checklist.

**Results:**

A total of 15 studies were included in this study. Prevalence of post-stroke depression in the studies varied from 24% to 90%. The pooled prevalence was 55% (95% CI 43%, 65%) with high heterogeneity (I^2^=94.83%). Prevalence also varied between the tools (HAMD -60%, GDS -70%, HADS -40%). The overall methodological quality of the included studies was very poor.

**Conclusion:**

It is evident from the meta-analysis that about half of those who survive a stroke experience post-stroke depression. The methods and tools used to investigate this was not rigorous and homogeneous. Hence results of this review imply the need to rigorously assess and effectively address post-stroke depression in India. Also, this review recommends future research to ensure methodological quality and generalizability of the study findings. This would help develop scalable, innovative public health intervention for post-stroke depression in the future.

## Background

During the past three decades, Stroke has been the second leading cause of death and disability worldwide. Every year about 5.5 million people are dying due to stroke globally [1]. There are about 80 million people who have survived a stroke and experience a substantial amount of disability post-stroke [2]. The Global Burden of Disease (GBD) study report that stroke and depression had been the major causes of total Years of Life Lost to disability (YLL) worldwide and depression is a common consequence of stroke [3]. About a third of the stroke survivors experience depression at any one time after their stroke [4]. Individuals with depression post-stroke are at a higher risk of delayed recovery, and poor quality of life [5]. Post-stroke depression is found to be associated with worse rehabilitation outcomes leading to increased disability in contrast to the patients with reduced/no post-stroke depression, where functional recovery was found to be better [6].

Much of the burden of stroke and post-stroke depression is borne by Low and Middle-income Countries (LMICs). As the second-most populous country in the world, India contributes about 28.5 million DALYs to the global stroke burden [7]. Post-stroke depression intensifies the overall outcomes of stroke among stroke survivors in India [8]. Recovery from functional impairment is expected to be lesser in post-stroke depressed patients compared to the non-depressed. There could be two reasons for this reflecting from a neuro-rehabilitation perspective. In most of the LMIC contexts including India, the focus of neurorehabilitation has been on the physical aspects of disability rather than a comprehensive holistic neurorehabilitation that aims to enable the affected individual to independently engage in everyday living [9]. It is believed that depression is a psychiatric disability, and it needs to be dealt with by the psychiatric rehabilitation team that may not be exclusively available in every rehabilitation facility. Even if it exists, they may not be linked or connected to the neuro-rehabilitation team [10].

The disabling experience of a stroke survivor with depression may have a negative impact that is far more than those stroke survivors without depression in this context. Stroke survivors in India are 3.4 times more likely to die during the first 10 years after stroke due to these reasons [11]. Subsequently, functional recovery, following stroke among those with depression could negatively impact their quality of life. It may also add up to an increase in mortality and greater chances of recurrent stroke [8].

Neurological rehabilitation for post-stroke depression is expected to be comprehensive and holistic [12]. Stroke results in various kinds of physical, mental, cognitive-perceptual, and socio-emotional impairments [13]. Hence rehabilitation post-stroke must encompass interventions that could address these diverse impairments and needs of the affected individuals [12]. Rehabilitation post-stroke must be viewed from the bio-psychosocial lens to enhance recovery and effective participation of the affected individual in society. Evidence for such comprehensive neurological rehabilitation for post-stroke depression that includes pharmacological and non-pharmacological interventions exist in Many High-Income Countries (HICs). However, this is of utmost public health importance in the context of LMICs where access to such rehabilitation services is very limited [13].

Early identification and diagnosis of post-stroke depression can prevent disability and improve rehabilitation. However, it is still unclear whether this evidence can be practically applied to the Indian context, especially given the out-of-pocket expenditure and non-availability of such services in the rural remote regions of the country [14]. Rehabilitation services are mostly provided by tertiary care hospitals, located in urban areas in India and poor people cannot afford to utilize such services. As a result of this, most of the stroke survivors resume their lives with disabilities post-stroke and these barriers act as a predisposing factor for post-stroke depression [15].

Existing literature either relates to stroke or depression separately in India. This diverts available resources for stroke and depression separately, thereby restricting efficient management of this double burden. They are also wide variation in the prevalence of post-stroke depression in India given the size, diverse lifestyle, and cultural practices of people in the country. Given these wide variations, it is very important to assess the true magnitude of the problem and understand the burden of post-stroke depression comprehensively. This could facilitate the development of effective and efficient public health interventions to meet the needs of those individuals experiencing post-stroke depression in India. Therefore, a systematic review was conducted.

### Description of the condition

“According to WHO, Stroke, the sudden death of some brain cells due to lack of oxygen when the blood flow to the brain is lost by blockage or rupture of an artery to the brain, is also a leading cause of dementia and depression [16]”. The effect of a stroke depends on the part of the brain being affected and its severity and occurrence of severe death could be due to sudden death. According to WHO, “Depression is a common mental disorder that presents with depressed mood, loss of interest or pleasure, decreased energy, feelings of guilt or low self-worth, disturbed sleep or appetite, and poor concentration [17]”. ‘The Diagnostic and Statistical Manual (DSM) IV categorizes post-stroke depression as “Mood disorder due to a general medical condition (i.e., stroke)” with the specifiers of depressive features, major depressive-like episodes, manic features, or mixed features [18]”.

### Why it is important to do this review

Worldwide researchers have made substantial efforts to estimate the prevalence of depression among post-stroke survivors, and highly varying estimates were mostly reported in many studies. Even in India, the literature is very limited on the prevalence and trends of post-stroke depression. While observational studies do provide a snapshot of the burden of stroke and depression associated with it, these studies do not give a detailed understanding of the patterns and dynamics of the rapidly increasing problem. The criteria used for assessing depression among post-stroke patients by the researchers were found to have a wide variation. Also, the degree to which depression is considered a problem among stroke survivors is vague. To assess the potential impact, there is a need for a robust estimate of post-stroke depression prevalence. The assessment will also help in highlighting treatment needs and will address the burden of stroke by framing a case for the development of the nationwide policy. Hence the present study aims to consolidate all the available literature based on the magnitude of depression among the stroke survivors in India and systematically review it.

## Objective

To estimate the prevalence of depression among stroke survivors in India

## Methods

### Criteria for considering *studies* for the review

#### Types of studies

In this review, all descriptive, observational, and prevalence studies were included. Studies like cross-sectional, case-control, and cohort that has possibilities to give prevalence information were also included in our initial search. Study designs other than observational or measured depression by proxy were excluded.

#### Types of participants

Participants who have experienced a stroke that is clinically diagnosed, namely ischemic, haemorrhagic, or transient ischemic attack (TIA) were included irrespective of age and gender.

#### Types of outcome measures

The outcome measure was the prevalence or frequency of depression among stroke survivors. Studies that report the prevalence of depression based on any criteria through any measurement techniques, as set by each study authors were included. The assessment carried by primary health physicians, specialist doctors, health workers either in a hospital or community setting was the target.

### Search strategy for the identification of studies

A Cochrane method of the literature search was carried out from 1960-2019 to identify all the published studies and unpublished studies with the English language. The electronic databases included in the search were PubMed-Medline, Global Health, HMIC Health Management Information Consortium, Journals@Ovid, PsycEXTRA, PsycINFO, LSHTM Journals@Ovid, Northern Light Life Sciences Conference Abstracts, Econlit, Embase Classic+Embase, Ovid MEDLINE(R), and Epub Ahead of Print, In-Process & Other Non-Indexed Citations, Daily and Versions (R, Social Policy and Practice) and GOOGLE SCHOLAR. For each electronic database, a search strategy specific to that database was developed. The search strategy used for various databases is given in appendix -I.

### Data collection and analysis

In this review, a step-by-step selection process was followed to select the studies for final inclusion.

#### Selection of studies

Firstly, the study title suitable for inclusion in the review was screened by two reviewers [AP, KN] independently. Those that were suitable were considered for the next stage of selection, and the remaining were excluded. We retrieved the abstracts of selected study titles and the same reviewers [AP, KN] independently scrutinized all selected abstracts. The full text of the articles included in the review was obtained and independently reviewed by the same authors. A third reviewer [SK] was available to arbitrate disagreement in the selection process.

#### Data extraction and management

A proforma for data extraction was developed for extracting data based on the selection criteria like author name, sample size, study setting, prevalence, and methods/techniques used to measure depression, age group, year of publication, and sampling method. The proforma was also pretested. One reviewer [SS] extracted the data from the selected full-text articles and it was cross-checked by a second reviewer [MD]. An expert’s consensus was drawn from a third reviewer [SK] if there were any disagreements.

#### Assessment of methodological quality of included studies

A nine-item tool was used to evaluate the methodological quality of studies based on the Joanna Briggs Institute Critical Appraisal Checklist [19] which includes aspects such as sampling frame, sampling technique, sample size, subjects and settings, data collection tools for assessment, etc. A color-coding in red, green, and yellow was carried out to indicate aspects that were not reported, reported and unclear respectively. One reviewer [MD] evaluated the data based on the items from the checklist and it was cross-checked by a second reviewer [SS]. Expert consensus was drawn from a third reviewer [SK] whenever necessary.

### Data Analysis

STATA version 14 with the “metaprop” [20] command was used for meta-analysis. The overall pooled prevalence of post-stroke depression and its confidence interval was computed. A biostatistician [MGL] analyzed the data and interpreted the findings.

#### Assessment of heterogeneity

Heterogeneity was assessed using Q statistics, degrees of freedom (df), and I2 statistic.

#### Assessment of reporting bias

A funnel plot and eggers test were performed to assess the publication bias.

#### Data synthesis (Meta-analysis)

The analysis was carried out with the assumption of a random effect model, which would give a more representative estimate than that of a fixed-effect model. Results are explained in terms of pooled prevalence, 95% confidence interval, and forest plot. Proportions are presented in the forest plots; however, all the results are presented in percentages for ease of understanding.

#### Subgroup analysis

Subgroup analysis was conducted based on tools, study design, and based on different states of India.

### Patient and Public Involvement

Patients and the public were not involved for the purpose of this systematic review.

### Ethics and Dissemination

Ethical approval for the study has been obtained from the independent institutional research ethics committee at the public health foundation of India (Indian Institute of Public Health) Hyderabad.

## Results

**Figure 1:**
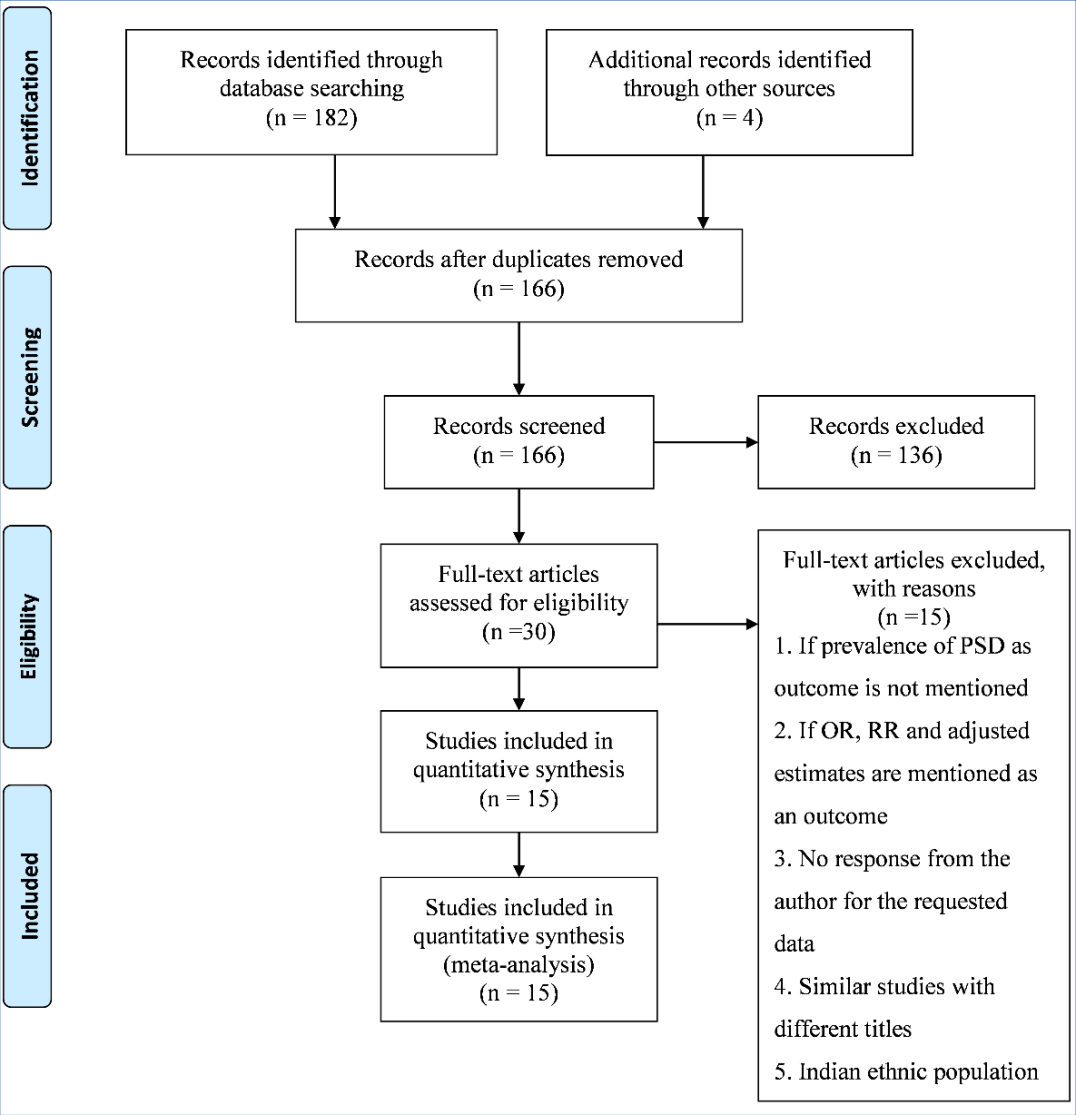
Prisma Flow Chart

The search yielded 186 records (electronic databases=182, handpicked=4). After removing the duplicates, 166 studies were eligible for screening. After screening for eligibility, 30 studies were identified as eligible for full-text screening. Out of 30, 15 studies did not meet the inclusion criteria. The reasons being studies not from India, and there was no information on the outcome of interest. In the present systematic review, only 15 studies fulfilled all criteria. The process of study selection is presented in the PRISMA flow chart (Figure 1).

### Study characteristics

#### Description of included studies

Fifteen studies met the inclusion criteria (Table 1), with a total of 1617 stroke survivors. Nine studies Rajashekaran et al. [21], Srivastava et al. [22], Chandran et al. [23], Patel et al. [24], Bonner et al. [25], Patel et al. [26], Saxena et al. [8], Isaac et al. [27], Rajkumar et al. [28] were cross-sectional, five studies Ghosal et al. [29], Paul et al. [30], Pandian et al. [31], Raju et al. [32], Rao et al. [33] were cohort and one study Singh et al. [34] did not provide information on the study design. The period of studies ranged from 2006 to 2018. Cross-sectional studies recruited patients who were either being treated in hospitals or were clinically or radiologically diagnosed or selected outside the neurological department or who are being treated in a rehabilitation centre. The recruitment of the participants suffering from astroke was based on the period ranging from a minimum of two weeks to not more than two years. In one of the cross-sectional studies Rajkumar et al. [28], participants were identified based on the computerized list and door-to-door survey, and all elderly were invited to participate. Four cohort studies recruited stroke patients who either had an episode of ischemic stroke or had a first-ever incident of stroke and one cohort study recruited stroke patients who completed more than one month of follow-up. Studies also selected cases within the community based on the WHO questionnaire and findings from the screening procedure. The minimum follow-up period of stroke survivors in all the five cohort studies was two weeks and the maximum till two years.

The included studies were from different parts of India. Six studies were conducted in the capital cities, two from West Bengal [29,30]; one study each from Karnataka [22], Telangana [33], Manipur [34], and Tamil Nadu [27]. One study was from Mangalore, Karnataka [21], and one was from Ludhiana, Punjab [32]. Three studies were conducted in the rural regions of India one study each from Kaniyambadi in Vellore [28], Cheroopa in Kozhikode [23], and Sewagram, Maharashtra [8]. Two studies were conducted in both urban and rural residences of Surendranagar, Gujarat [24,26], and there were two multi-centered studies [25,31].

The number of stroke patients in the 15 studies ranged from 32 to 1000 (Mean=180). Five studies included patients with infarct and hemorrhagic stroke [8,22,23,29,30], whereas two studies included patients with ischemic and hemorrhagic stroke [26,32]. Likewise, four studies included patients with stroke based on lesion location [21,24,31,33]. Two studies included patients with stroke based on Transient Ischemic attack and ischemia, respectively [25,28]. Two studies did not mention what types of patients were included [27,34]. Age ranged from 20 to 81 years. One study from Tamil Nadu included patients of the geriatric age group (50 to 90 years). Prevalence of post-stroke depression based on gender was not mentioned in any of the studies.

#### Scalesfor assessing depressive symptoms

Six screening scales were used by the included study authors to assess depressive symptoms among stroke survivors. Four studies Srivastava et al. [22], Patel et al. [24], Patel et al. [26], Isaac et al. [27] used the Hamilton Rating Scale for Depression (HAM-D), two studies Ghosal et al. [29], Paul et al. [30] used the Geriatric Depression Scale (GDS). One study each used the Beck Depression Inventory (BDI), Montgomery-Asberg Depression Rating Scale (MADRS), and PSE & diagnosed based DSM-IV. Two studies Bonner et al. [25], Raju et al. [32] used the Hospital Anxiety and Depression Scale (HADS), and one study Singh et al. [34] used the Geriatric mental scale (GMS). There was no mention of the type of scales used in one of the studies Pandian et al. [31]. Few of the studies Rajashekaran et al. [21], Rao et al. [33] used multiple scales to assess depression.

Out of the 15 studies, six studies Rajashekaran et al. [21], Srivastava et al. [22], Patel et al. [24], Saxena et al. [8], Paul et al. [30], Rao et al. [33] exclusively estimated the prevalence of post-stroke depression. Three studies Patel et al. [26], Raju et al. [32], Singh et al. [34], estimated depression and anxiety and other psychiatric morbidities among stroke survivors, and six studies Chandran et al. [23], Bonner et al. [25], Isaac et al. [27], Rajkumar et al. [28], Ghosal et al. [29], Pandian et al. [31], included depression as one of the associated factors among stroke survivors.

### Methodological quality

JBI critical appraisal checklist [19] was used to evaluate the methodological quality of studies. All the 15 included studies had a low risk of bias (100%) based on sampling frame as it were either hospital setting, Stroke rehabilitation Centre or from the secondary data. The sample size was inadequate and the participants’ sampling strategy was inappropriate and leads to a high risk of bias in 73.3% and 80% of the studies respectively. The study subject and the setting were clearly described in most of the studies (93.3%). Low risk of bias was observed in 93.3% of the studies which used valid and reliable tools to assess the identified conditions and only 13.3% of studies had data analysis with sufficient coverage of identified sample. One-third of the studies used standard criteria to measure the conditions and statistical analysis was unclear in the same proportion of the studies. The response rate was unclear and not managed appropriately in 80% of the studies.

**Figure 2:**
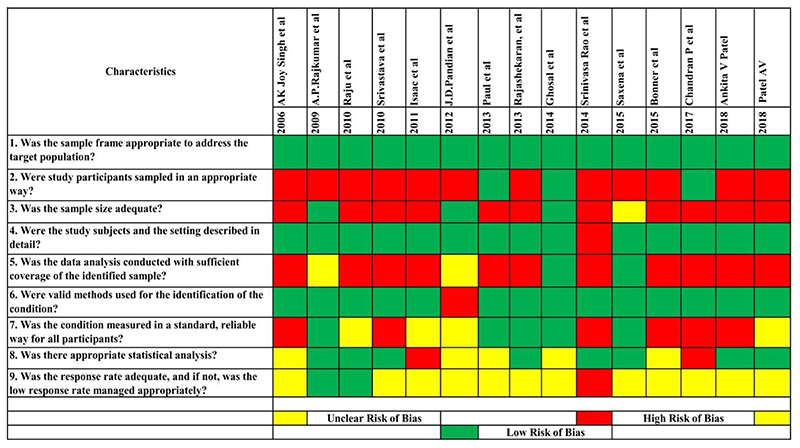
Study wise Quality Assessment of Included Studies based on JBI Critical Appraisal Checklist

**Figure 3:**
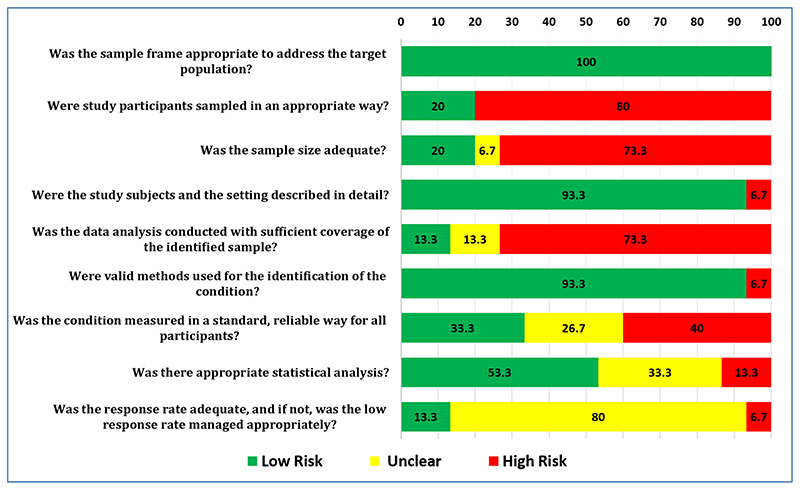
Component wise Methodological Quality Assessment of Included Studies based on Cochrane guidelines

Figure 2 provides the quality assessment carried for the included studies. Figure 3 displays a graph indicating the percentage of methodological quality assessed for each item, which also represents that the overall quality of the studies was poor.

### Effects

#### Prevalence of Depression among Stroke Survivors

**Figure 4:**
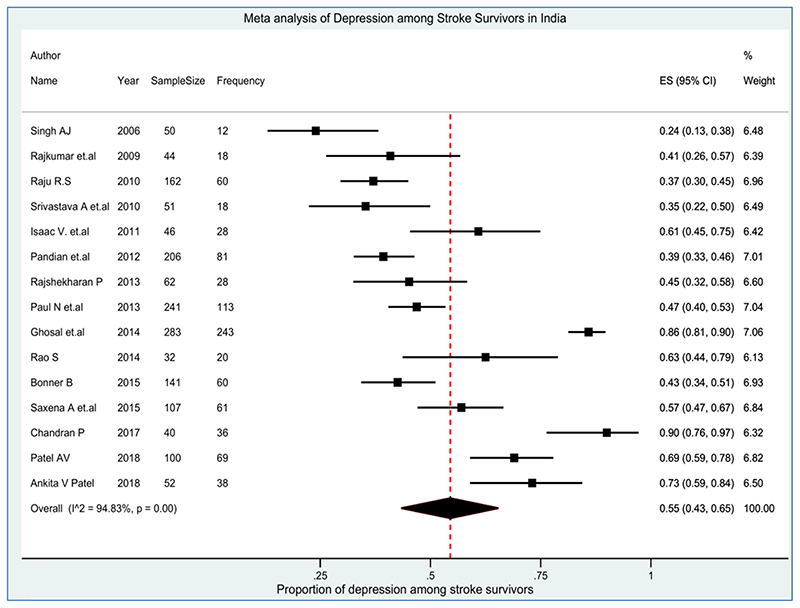
Pooled Prevalence of Depression among Stroke Survivors in India

All the included studies reported the prevalence of post-stroke depression and the overall pooled prevalence of depression among stroke survivors (N=1617) was 55% (95% CI 43%, 65%) with significantly high heterogeneity (Q=270.57, df=14, *p*<0.001, I^2^=94.83%) (Figure 4). Out of 15 included studies, five cohort studies provided higher (58% (95% CI 46%, 69%)) prevalence of depression compared to nine cross-sectional studies (55% (95% CI 33%, 76%) and study design were not reported in one of the studies. The prevalence of depression was higher in West Bengal with a pooled estimate of 74% (95% CI 71%, 78%), followed by Gujarat 70% (95% CI 63%, 78%), Tamil Nadu 51% (41%, 61%) and Karnataka 41% (32%, 50%).

**Figure 5:**
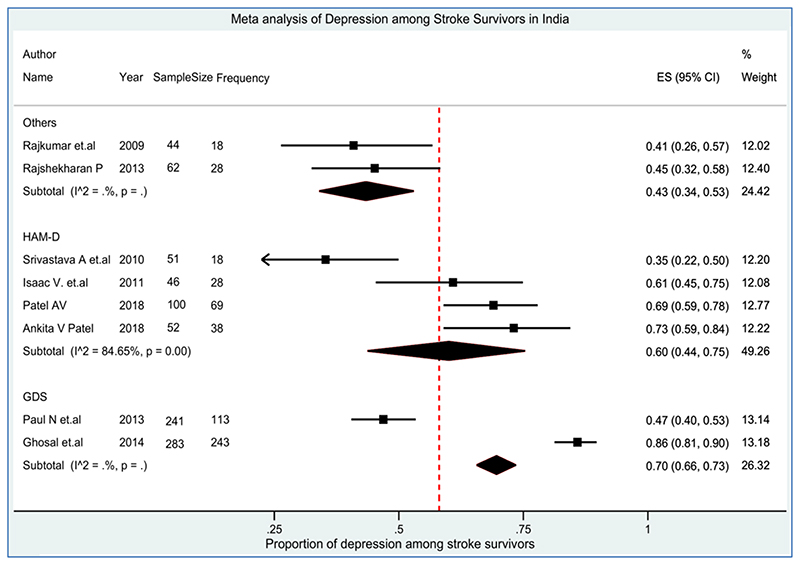
Prevalence of Depression among Stroke Survivors: Based on Tools

There was wide variability in the estimates among the tools adopted by the authors of the included studies. The highest prevalence (90%) was seen in a study that used the BDI scale. Likewise, the prevalence of depression was 70% based on the GDS scale (two studies), 60% based on HAM-D (four studies), 57% based on the MADRS scale, and 40% based on the HADS scale (two studies). The results are displayed in Table 2. Also Figure 5 demonstrated an asymmetrical funnel plot for the prevalence of depression in stroke survivors, suggesting evidence of publication bias although quantitative testing with Egger bias confirmed this was not significant (intercept=0.601, *p*=0.713).

Additional figures from the meta-analysis by sub-groups were provided as supplementary figures 1–3.

## Discussion

Evidence from this review shows that the prevalence of post-stroke depression in India is about 55%. Given the methodological quality of the studies available on this topic, the prevalence ranged from 24% to 90%. However, the result from this meta-analysis has to be taken seriously because the stroke has been the second leading cause of death and disability globally and also in India for the past two decades. Post-Stroke Depression is therefore an important public health problem in India. Currently, there are three national programs in India directly related to Post-Stroke Depression. The national program on Non-Communicable diseases (NPCDCS), the National mental health program (NMHP), and the National program for the healthcare of the elderly (NPHCE) [35–38]. However, none of these programs have specific operational strategies within their implementation plans to look at Post-Stroke Depression exclusively or even within their general operational strategies of the programs. Though most of these programs look at a stroke and its management from a medical perspective, it is high time that these programs consider medical management of Post-Stroke Depression at the least. Let alone the rehabilitation aspects.

Post-stroke depression requires a combination of therapeutic strategies such as medical, behavioral, and social models of care [39]. This kind of neuro-rehabilitation can optimize the advantages of neuromodulation for enhancing functional independence and social participation of the affected individual [40]. The advantages of using combined multi-dimensional strategies have been found to outweigh the specific, standalone strategies for managing post-stroke depression [40]. This is especially critical because Post-Stroke Depression adversely affects the quality of life of individuals experiencing stroke and also their families. The disabling effects of Post-Stroke Depression shackle the prognosis of the stroke survivor receiving high-intensity rehabilitation too. The results of this review thus are very pertinent to the development of specific treatment pathways, practice guidelines, and management strategies of Post-Stroke Depression either within the national programs or outside their boundaries. It is also very important to bridge the gaps in evidence on the burden of Post-Stroke Depression and its management nationally with well-designed methodologically rigorous studies in the future. This would enable the needs of the stroke survivors with Post-Stroke Depression and their families to be addressed with effective evidence-based interventions in the near future.

### Strengths and limitations of this study

- More than five reviewers were involved in actual screening at various stages to identify the studies to be included systematically.
- The methodological quality of the included studies was rigorously assessed using a mix of JBI Critical Appraisal Checklist and Cochrane Guidelines.
- The review was restricted to only India.
- Meta-analysis was done for descriptive reasons although included studies were heterogeneous.

## Supplementary Material

Figure 1-3 and Search Strategy

## Figures and Tables

**Table 1:** Description of studies included in the review

Study period	State	Type of patients	Mean age/Age Group (in years)	Study Design	Type of Scale	Total number of patients	Patients with depression	Reference
2006	Manipur	Post-Stroke Hemiplegic	No information	No information	PSE & diagnosed based DSM-IV	50	12	[34]
2009	Tamil Nadu	Elderly: Transient Ischemic Attack	72.54 ± 5.87 (66.67, 78.41)	Cross Sectional study	GMS	44	18	[28]
2010	Punjab	Stroke: Ischemic, Hemorrhagic	54.3 ± 12.9 (41.4, 67.2)	Prospective hospital-based study	HADS	162	60	[32]
2010	Karnataka	Stroke: Infarct, Hemorrhagic	46.06 ± 11.19 (34.87, 57.25)	Cross-Sectional Study	HAM-D	51	18	[22]
2011	Tamil Nadu	Stroke	63 ± 7.2 (55.8, 70.2)	Cross-sectional study	HAM-D	46	28	[27]
2012	Multi-center: Andhra Pradesh Kerala, Punjab	Stroke: Based on lesion location (OSCP)	58.1 ± 13.7 (44.4, 71.8)	Prospective multicenter study	No information	206	81	[31]l
2013	West Bengal	Stroke: Infarct, Hemorrhage	62.7 ± 13.04 (49.66, 75.74)	Cohort study	GDS	241	113	[30]l
2013	Karnataka	Stroke: Based on lesion location	57.89 ± 7.12 (50.77, 65.01)	Cross-sectional study	BDI, MADRS	62	28	[21]
2014	West Bengal	Stroke: Infarct, Hemorrhage, mixed	64.27 ± 13.08 (51.19, 77.35)	Cohort study	GDS	283	243	[29]
2014	Telangana	Stroke: Based on laterality of lesion	(20, 64)	Prospective Study	HAM-D, MADRS	32	20	[33]
2015	Maharashtra	Stroke: Infarct, Hemorrhagic	59.13 ± 11.66 (47.47, 70.79)	Cross Sectional study	MADRS	107	61	[8]
2016	Kerala, Punjab	Stroke: Ischemic	48 ± 8.8 (39.2, 56.8)	Cross Sectional study	HADS	141	60	[25]
2017	Kerala	Stroke: Infarct, Hemorrhagic	70.58 ± 10.7 (59.88, 81.28)	Cross Sectional study	BDI	40	36	[23]
2018	Gujarat	Stroke: Ischemic, Hemorrhagic	(21, 80)	Cross Sectional study	HAM-D	100	69	[24]
2018	Gujarat	Stroke: Based on lesion	(20, 65+)	Cross Sectional study	HAM-D	52	38	[26]

**Table 2:** Subgroup Analysis Based on Tools, Study Design and States

Subgroups	Characteristics	Number of studies	Number of participants (n)	Pooled prevalence (95% CI)	Q	Df	I^2^
**Based on study design**	Cross-sectional	9	643	58% (46%,69%)	64.22	8	87.54%
	Cohort	5	924	55% (33%,76%)	184.32	4	97.83%
**Based on states**	Tamil Nadu	2	90	51% (41%,61%)	-	1	-
	Karnataka	2	113	41% (32%,50%)	-	1	-
	West Bengal	2	524	74% (71%,78%)	-	1	-
	Gujarat	2	152	70% (63%,78%)	-	1	-
**Based on tools**	HAM-D	4	249	60% (44%,75%)	19.54	3	84.65%
	GDS	2	524	70% (66%,73%)	-	1	-
	HADS	2	303	40% (34%,45%)	-	1	-
	BDI and MADRS	1	62	45% (32%,58%)	-	-	-
	PSE & diagnosed based DSM-IV	1	50	24% (13%,38%)	-	-	-
	BDI	1	40	90% (76%,97%)	-	-	-
	HAM-D and MADRS	1	32	63% (44%,79%)	-	-	-
	GMS	1	44	41% (26%,57%)	-	-	-
	MADRS	1	107	57% (47%,67%)	-	-	-
	No information	1	206	39% (33%,46%)	-	-	-

## Abbreviations

HAM-D: Hamilton Depression Rating Scale
MADRS: Montgomery Asberg Depression Rating Scale
HADS: Hospital Anxiety and Depression Scale
BDI: Beck Depression Inventory
GDS: Geriatric Depression Scale
GMS: Geriatric Mental Scale
PSE: Present State Examination
DSM: Diagnostic and Statistical Manual of Mental Disorders
GBD: Global Burden of Disease
YLL: Years of Life Lost
DALYs: Disability Adjusted Life Years
LMICs: Lower Middle-Income Countries
WHO: World Health Organization
TIA: Transient Ischaemic Attack
HMIC: Health Management Information Consortium
LSHTM: London School of Hygiene and Tropical Medicine
DF: Degree of Freedom
PRISMA: Preferred Reporting Items for Systematic Review and Meta-analysis
JBI: Joanna Briggs Institute
PHFI: Public Health Foundation of India
NPCDCS: National Program for Prevention and Control of Cancer, Diabetes, Cardiovascular diseases, and Stroke
NMHP: National Mental Health Program
