# Supplementary material for: Prevalence of Depression among Stroke Survivors in India: A Systematic Review and Meta-Analysis
Source: Front Neurol Neurosci Res. Author manuscript; Available in PMC 2021 Jun 24. (PMC7611044; doi:10.51956/FNNR.100008)
Supplement: Figure 1-3 and Search Strategy [file EMS127706-supplement-Figure_1_3_and_Search_Strategy.pdf]

Frontiers in Neurology and Neuroscience Research
Volume 2
Article ID: 100008
DOI: 10.51956/FNNR.100008

Research Article

Prevalence of Depression among Stroke Survivors in India: A Systematic Review and Meta-Analysis

Abhilash Patra[1], Karakapatla Nitin[1], Ng Marina Devi[1], Srinivasan Surya[1], Melissa G. Lewis[1] and Sureshkumar Kamalakannan[1*]

[1]Indian Institute of Public Health, Kaloji Narayana Rao University of Health Sciences, India

***Corresponding authors**: Sureshkumar Kamalakannan, India Alliance DBT- Wellcome Trust Fellow, Indian Institute of Public Health, Kaloji Narayana Rao University of Health Sciences, Plot No: 1, ANV Arcade, Amar Cooperative Society, Kavuri Hills, Madhapur, Telangana, India, Tel: +91 9676333412, +44 7366962444; E-mail: suresh.kumar@iiphh.org

Received: February 09, 2021; **Accepted**: April 07, 2021; **Published**: April 14, 2021

Copyright: ©2021 Patra A. This is an open access article distributed under the Creative Commons Attribution License, which permits unrestricted use, distribution, and reproduction in any medium, provided the original work is properly cited.

Supplementary Figures

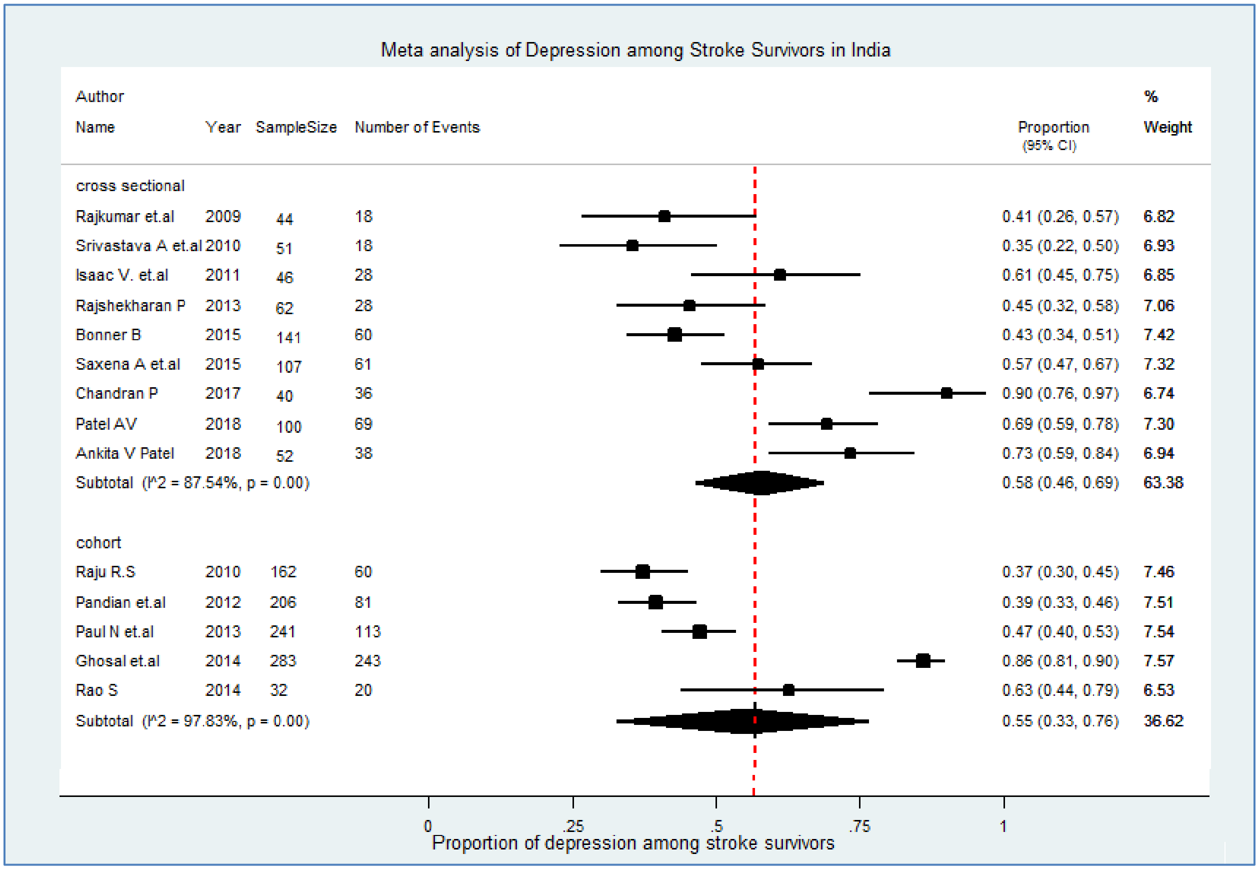

Supplementary Figure 1. Prevalence of Depression among Stroke Survivors: Based on Study Design

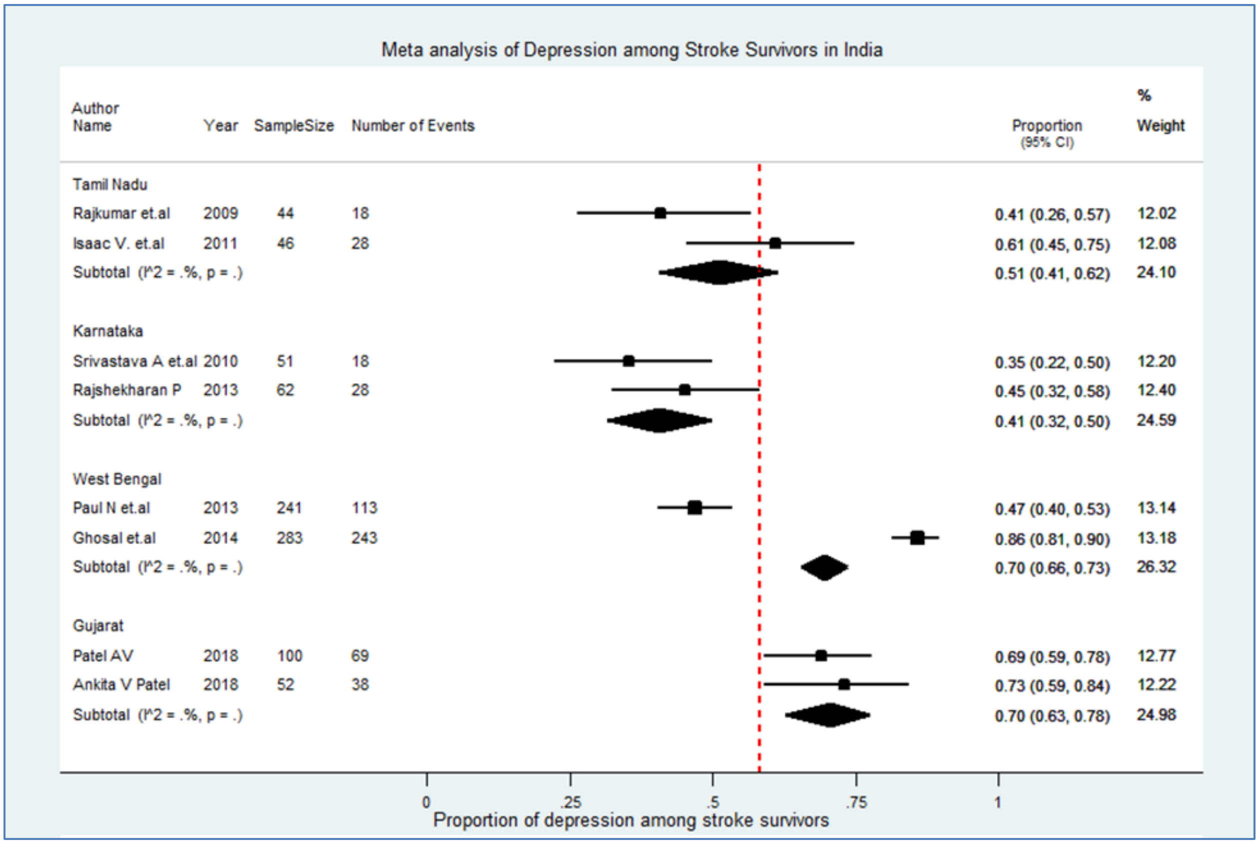

Supplementary Figure 2. Prevalence of Depression among Stroke Survivors: Based on States

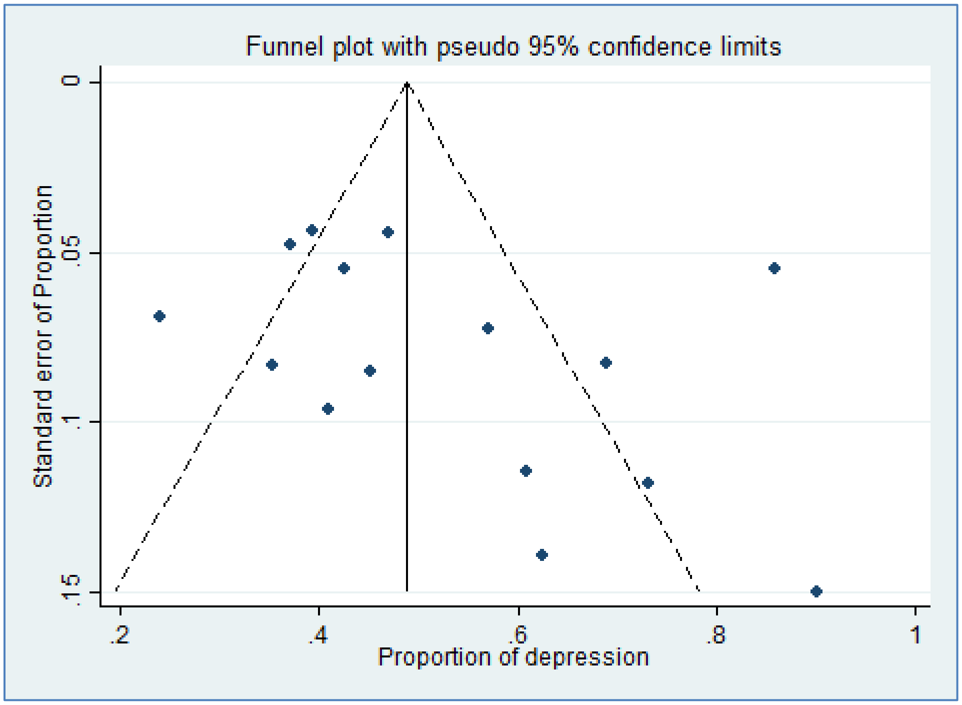

Supplementary Figure 3. Prevalence of Depression among Stroke Survivors: Funnel Plot

Appendix-I

Search strategy

Database: Global Health <1910 to 2019 Week 21>, HMIC Health Management Information Consortium <1979 to April 2019>, Journals@Ovid Full Text <May 31, 2019>, PsycEXTRA <1908 to May 13, 2019>, PsycINFO <1806 to May Week 4 2019>, LSHTM Journals@Ovid, Northern Light Life Sciences Conference Abstracts <2010 - 2019 Week 21>, Econlit <1886 to May 23, 2019>, Embase Classic+Embase <1947 to 2019 May 31>, Ovid MEDLINE(R) and Epub Ahead of Print, In-Process & Other Non-Indexed Citations, Daily and Versions(R) <1946 to May 31, 2019>, Social Policy and Practice <201904>

1. Depression.mp. [mp=ab, ti, ot, bt, hw, id, cc, tx, ct, sh, tc, tm, tn, dm, mf, dv, kw, fx, dq, nm, kf, ox, px, rx, an, ui, sy, pt] (1923012)
2. Depressive Disorder.mp. [mp=ab, ti, ot, bt, hw, id, cc, tx, ct, sh, tc, tm, tn, dm, mf, dv, kw, fx, dq, nm, kf, ox, px, rx, an, ui, sy, pt] (252659)
3. Adjustment Disorders.mp. [mp=ab, ti, ot, bt, hw, id, cc, tx, ct, sh, tc, tm, tn, dm, mf, dv, kw, fx, dq, nm, kf, ox, px, rx, an, ui, sy, pt] (10098)
4. (depress* or melanchol* or (adjustment or reactive or dysthymic)).mp. [mp=ab, ti, ot, bt, hw, id, cc, tx, ct, sh, tc, tm, tn, dm, mf, dv, kw, fx, dq, nm, kf, ox, px, rx, an, ui, sy, pt] (4669912)
5. 1 or 2 or 3 or 4 (4669912)
6. Epidemiologic Studies.mp. [mp=ab, ti, ot, bt, hw, id, cc, tx, ct, sh, tc, tm, tn, dm, mf, dv, kw, fx, dq, nm, kf, ox, px, rx, an, ui, sy, pt] (132772)
7. exp Case-Control Studies/ (1169635)
8. exp Cohort Studies/ (2381457)
9. Cross-Sectional Studies/ (469058)
10. (epidemiologic adj (study or studies)).ab,ti. (78012)
11. case control.ab,ti. (358846)
12. (cohort adj (study or studies)).ab,ti. (606016)
13. cross sectional.ab,ti. (1014637)
14. cohort analy$.ab,ti. (25475)
15. (follow up adj (study or studies)).ab,ti. (154194)
16. longitudinal.ab,ti. (794064)
17. retrospective$.ab,ti. (2150060)
18. prospective$.ab,ti. (2126873)
19. (observ$ adj3 (study or studies)).ab,ti. (576757)
20. adverse effect?.ab,ti. (442419)
21. 6 or 7 or 8 or 9 or 10 or 11 or 12 or 13 or 14 or 15 or 16 or 17 or 18 or 19 or 20 (8245352)
22. cerebrovascular disorders/ (83424)
23. exp basal ganglia cerebrovascular disease/ (1285)
24. exp brain ischemia/ (278800)
25. exp carotid artery diseases/ (116041)
26. stroke/ (282867)
27. exp brain infarction/ (109570)
28. exp cerebrovascular trauma/ (52240)

29. hypoxia-ischemia, brain/ (7442)
30. exp intracranial arterial diseases/ (67913)
31. exp intracranial arteriovenous malformations/ (17012)
32. exp "Intracranial Embolism and Thrombosis"/ (525189)
33. exp intracranial hemorrhages/ (213369)
34. vasospasm, intracranial/ (7490)
35. vertebral artery dissection/ (6117)
36. aneurysm, ruptured/ and exp brain/ (1461)
37. brain injuries/ (105287)
38. brain injury, chronic/ (78406)
39. (stroke or poststroke or post-stroke or cerebrovasc$ or brain vasc$ or cerebral vasc$ or cva$ or apoplex$ or isch?emi$ attack$ or tia$1 or neurologic$ deficit$ or SAH or AVM).tw. (1469975)
40. ((brain$ or cerebr$ or cerebell$ or cortical or vertebrobasilar or hemispher$ or intracran$ or intracerebral or infratentorial or supratentorial or MCA or anterior circulation or posterior circulation or basal ganglia) adj5 (isch?emi$ or infarct$ or thrombo$ or emboli$ or occlus$ or hypox$ or vasospasm or obstruction or vasculopathy)).tw. (493976)
41. ((lacunar or cortical) adj5 infarct$).tw. (25939)
42. ((brain$ or cerebr$ or cerebell$ or intracerebral or intracran$ or parenchymal or intraventricular or infratentorial or supratentorial or basal gangli$ or subarachnoid or putaminal or putamen or posterior fossa) adj5 (haemorrhage$ or hemorrhage$ or haematoma$ or hematoma$ or bleed$)).tw. (305387)
43. ((brain or cerebral or intracranial or communicating or giant or basilar or vertebral artery or berry or saccular or ruptured) adj5 aneurysm$).tw. (113019)
44. (vertebral artery dissection or cerebral art$ disease$).tw. (4278)
45. ((brain or intracranial or basal ganglia or lenticulostriate) adj5 (vascular adj5 (disease$ or disorder or accident or injur$ or trauma$ or insult or event))).tw. (9236)
46. ((isch?emic or apoplectic) adj5 (event or events or insult or attack$)).tw. (160100)
47. ((cerebral vein or cerebral venous or sinus or sagittal) adj5 thrombo$).tw. (26851)
48. ((intracranial or cerebral art$ or basilar art$ or vertebral art$ or vertebrobasilar or vertebral basilar) adj5 (stenosis or isch?emia or insufficiency or arterioscler0$ or atheroscler0$ or occlus$)).tw. (92706)
49. ((venous or arteriovenous or brain vasc$) adj5 malformation$).tw. (56648)
50. ((brain or cerebral) adj5 (angioma$ or hemangioma$ or haemangioma$)).tw. (3343)
51. exp aphasia/ or anomia/ or hemiplegia/ or hemianopsia/ or exp paresis/ or deglutition disorders/ or dysarthria/ or pseudobulbar palsy/ or muscle spasticity/ (213869)
52. (aphasi$ or apraxi$ or dysphasi$ or dysphagi$ or deglutition disorder$ or swallow$ disorder$ or dysarthri$ or hemipleg$ or hemipar$ or paresis or paretic or hemianop$ or hemineglect or spasticity or anomi$ or dysnomi$ or acquired brain injur$ or hemiball$).tw. (442392)
53. ((unilateral or visual or hemispatial or attentional or spatial) adj5 neglect).tw. (13427)
54. 22 or 23 or 24 or 25 or 26 or 27 or 28 or 29 or 30 or 31 or 32 or 33 or 34 or 35 or 36 or 37 or 38 or 39 or 40 or 41 or 42 or 43 or 44 or 45 or 46 or 47 or 48 or 49 or 50 or 51 or 52 or 53 (3201412)
55. 5 or 21 or 54 (14340954)
56. 5 and 21 and 54 (99872)

57. limit 56 to "300 adulthood <age 18 yrs and older>" [Limit not valid in Global Health,HMIC,Journals@Ovid,Your Journals@Ovid,Northern Light Life Sciences Conference Abstracts,Econlit,Embase,Ovid MEDLINE(R),Ovid MEDLINE(R) Daily Update,Ovid MEDLINE(R) In-Process,Ovid MEDLINE(R) Publisher,Social Policy and Practice; records were retained] (99522)
58. limit 57 to english [Limit not valid in Northern Light Life Sciences Conference Abstracts,Social Policy and Practice; records were retained] (98219)
59. limit 58 to yr="1990 -Current" (97597)
60. exp India/ (365011)
61. 56 and 57 and 58 and 59 and 60 (162)
62. 59 and 60 (162)
63. remove duplicates from 62 (132)
